# Combined Antibacterial Effects of Goat Cathelicidins With Different Mechanisms of Action

**DOI:** 10.3389/fmicb.2018.02983

**Published:** 2018-11-30

**Authors:** Pavel V. Panteleev, Ilia A. Bolosov, Alexander À. Kalashnikov, Vladimir N. Kokryakov, Olga V. Shamova, Anna A. Emelianova, Sergey V. Balandin, Tatiana V. Ovchinnikova

**Affiliations:** ^1^M.M. Shemyakin and Yu. A. Ovchinnikov Institute of Bioorganic Chemistry, Russian Academy of Sciences, Moscow, Russia; ^2^Institute of Experimental Medicine, Saint Petersburg, Russia

**Keywords:** antimicrobial peptide, cathelicidin, goat, proline-rich peptide, synergy, extensively drug-resistant, immune system

## Abstract

Being essential components of innate immune system, animal antimicrobial peptides (AMPs) also known as host-defense peptides came into sharp focus as possible alternatives to conventional antibiotics due to their high efficacy against a broad range of MDR pathogens and low rate of resistance development. Mammalian species can produce a set of co-localized AMPs with different structures and mechanisms of actions. Here we examined the combined antibacterial effects of cathelicidins, structurally diverse family of host-defense peptides found in vertebrate species. As a model we have used structurally distinct cathelicidins expressed in the leukocytes of goat *Capra hircus*. The recombinant analogs of natural peptides were obtained by heterologous expression in bacterial system and biological activities as well as the major mechanisms of antibacterial action of the peptides were investigated. As the result, the marked synergistic effect against wide panel of bacterial strains including extensively drug-resistant ones was observed for the pair of membranolytic α-helical amphipathic peptide ChMAP-28 and Pro-rich peptide mini-ChBac7.5Nα targeting a bacterial ribosome. ChMAP-28 was shown to damage the outer bacterial membrane at sub-inhibitory concentrations that could facilitate Pro-rich peptide translocation into the cell. Finally, resistance changes under a long-term continuous selective pressure of each individual peptide and the synergistic combination of both peptides were tested against *Escherichia coli* strains. The combination was shown to keep a high activity after the 26-days selection experiment in contrast to mini-ChBac7.5Nα used alone and the reference antibiotic polymyxin B. We identified the point mutation leading to amino acid substitution V102E in the membrane transport protein SbmA of the mini-ChBac7.5Nα-resistant strain obtained by selection. The experiments revealed that the presence of sub-inhibitory concentrations of ChMAP-28 restored the activity of mini-ChBac7.5Nα against this strain and clinical isolate with a weak sensitivity to mini-ChBac7.5Nα. The obtained results suggest a potential medical application of synergistic combinations of natural cathelicidins, which allows using a lower therapeutic dose and minimizes the risk of resistance development.

## Introduction

Over recent years, a growing number of bacterial species became resistant to clinically significant antibiotics. Host defense antimicrobial peptides (AMPs) came into sharp focus as possible alternatives to conventional antibiotics due to their high efficacy against a broad range of multiple drug-resistant pathogens, a rapid membranolytic mode of action and, as consequence, a low risk of resistance development. Cathelicidins, one of the major groups of animal AMPs, are known to be the key molecular factors of innate immunity of most vertebrate species, from hagfish to human (Kościuczuk et al., 2012). Along with direct antimicrobial action, these peptides possess immunomodulatory activities, such as inhibition of apoptosis, cytokine stimulating, lipopolysaccharide (LPS) neutralizing, promotion of wound healing, and regulation of adaptive immune responses. All the above suggest that these compounds can be prototypes for novel therapeutics with complex mechanism of action (Steinstraesser et al., 2011). The precursors of cathelicidins are produced in immune and epithelia cells and contain the N-terminal part of 99–114 amino acid residues which is known as the cathelin domain. This structure is highly conserved among vertebrates, whereas the C-terminal domain, encoding the mature peptide, shows substantial heterogeneity. Interestingly, the cathelin domain does not exhibit a protease inhibitory function regardless of its high structural similarity to cystatins (Pazgier et al., 2013). Therefore, the question why the cathelin domain is highly conserved among vertebrate cathelicidins is still open. It is believed that the precursor proteins could play a role in the secretion, intracellular trafficking as well as prevent cytotoxicity of mature peptides and their proteolytic degradation. The potential toxicity of cathelicidins is also controlled by their compartmentalization in cytoplasmic granules of immune cells. In case of contact with pathogens AMPs are activated by fusion of procathelicidin-containing specific granules (or large granules of ruminant neutrophils) with the elastase/proteinase 3-containing azurophil granules and either the cytoplasmic membrane or phagosome (Graf et al., 2017). Secondary structures of mature cathelicidins include α-helices, β-hairpins, and extended linear regions enriched with Trp or Pro residues. Interestingly, neutrophils of some artiodactyls, including goats, do not contain defensin-like AMPs (Zhao et al., 1999), suggesting a key role of cathelicidins in the protection of these animals against pathogens. Study of artiodactyl cathelicidins can provide new molecular insight into their role in the host defense.

**FIGURE 1:**
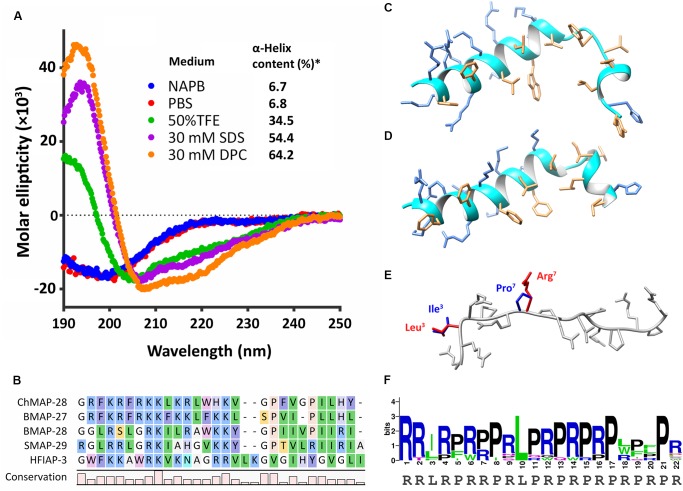
Structure analysis of goat cathelicidins. **(A)** CD-spectra of ChMAP-28 in 10 mM sodium phosphate buffer (NAPB, pH 7.4), phosphate-buffered saline (PBS, pH 7.4), 50% TFE, 30 mM SDS micelles, 30 mM DPC micelles. ^∗^The CONTINLL program (Provencher and Glöckner, 1981) was used for data analysis. **(B)** Alignment of the mature ChMAP-28 with α-helical bovine, sheep, and hagfish cathelicidins. **(C)** Spatial structure of ChMAP-28 was simulated in the MODELLER software (Sali and Blundell, 1993) by homology modeling on the basis of the NMR structure **(D)** of BMAP-27 (PDB 2KET) serving as a template. **(E)** Spatial structure of the mini-ChBac7.5Nα(1–16) fragment was modeled and overlaid on the basis of the crystal structure of the Bac7(1–16) bound to bacterial 70S ribosome (PDB 5F8K). Varying residues are marked with red for goat cathelicidin and blue for bovine cathelicidin. The structures were visualized by the Chimera software (Pettersen et al., 2004). **(F)** Amino acid frequency in mini-ChBac7.5Nα and its orthologs from mammalian species. The graph was plotted using the WebLogo server.

A number of studies on the synergy between AMPs and conventional antibiotics have been performed over the last years (Cassone and Otvos, 2010; Reffuveille et al., 2014; Simonetti et al., 2014; Ribeiro et al., 2015; de la Fuente-Núñez et al., 2016; Lázár et al., 2018). In contrast, the synergy between AMPs is not well investigated although this phenomenon might contribute to understanding of substantial peptide diversity at any host anatomic site. In most cases the repertoire of structurally diverse animal AMPs make possible both disturbing the membrane integrity of pathogenic microorganisms and inhibiting a number of metabolic processes via interaction with intracellular targets. Such a complex mechanism of action appears to prevent the development of resistance to AMPs. The present work is aimed to examine combined antibacterial effects of structurally distinct cathelicidins expressed in leukocytes of the domestic goat *Capra hircus*. Previously, we have isolated two novel AMPs mini-bactenecins, designated as mini-ChBac7.5Nα and mini-ChBac7.5Nβ, from leukocytes of the domestic goat (Shamova et al., 2016). These peptides are N-terminal fragments (22 and 21 aa, respectively) of the hypothetic ChBac7.5 peptide also classified as cathelicidin-3. Being Pro-rich AMPs, mini-bactenecins are thought to target intracellular structures such as the 70S ribosome and/or heat shock protein DnaK (Graf et al., 2017). In the study, we investigated a biological significance of the PRPRPR fragment localized at the C-terminus of mini-ChBac7.5Nα. For this purpose, a comparative testing of the wild-type peptide and its short derivative termed as mini-ChBac7.5Nα(1–16) was carried out. Earlier, bovine Bac7(1–16) was shown to be the minimal fragment of the native 60-residue peptide Bac7 displaying both antimicrobial activity in broth microdilution tests and ability to inhibit protein synthesis *in vitro* (Benincasa et al., 2004; Seefeldt et al., 2016). Along with mini-ChBac7.5Nα, the previously not investigated *C. hircus* myeloid AMP cathelicidin-6, designated as ChMAP-28, was chosen as the second component of the model system. The peptide primary structure was deduced from the deposited in GenBank mRNA sequence (AJ243126.1) coding the appropriate precursor protein. The novel cathelicidin has relatively high homology with the α-helical bovine cathelicidin BMAP-27 (Figure 1). ChMAP-28 contains eleven basic amino acid residues (Arg, Lys, His). As goat leukocytes were shown to simultaneously express mRNA for both cathelicidin-3 and -6 (Zhang et al., 2014), we supposed that the peptides were co-localized in the cells and could act synergistically during the immune response. The combined antibacterial effects of the goat cathelicidins were studied by a checkerboard titration method against a set of bacterial strains including the “ESKAPE” pathogens. The role of each cathelicidin in the synergistic cooperation and their predominant mechanisms of action were elucidated. Finally, antibacterial activity changes under a long-term continuous selective pressure of the individual peptides and their combination were investigated against *Escherichia coli* strains.

## Materials and Methods

All the bacterial strains used in this study are listed in Table 1. The clinical isolates were collected and provided by Sechenov First Moscow State Medical University hospital and Solixant LLC (Moscow, Russia). The resistant to conventional antibiotics strains were defined as extensively drug resistant (XDR) according to (Magiorakos et al., 2012).

**Table 1:** Bacterial strains used in this study.

Bacterial strain	Characteristics (source, antibiotic resistance)
*Micrococcus luteus* B-1314	Laboratory strain (VKM collection)
*Bacillus subtilis* B-886	Laboratory strain (VKM collection)
*Enterococcus faecalis* ATCC 29212	Laboratory strain (ATCC collection)
*Staphylococcus aureus* ATCC 29213	Laboratory strain (ATCC collection)
*Staphylococcus aureus* 209P	Laboratory strain (ATCC collection)
*Escherichia coli* DH10B	Cloning strain (Invitrogen)
*Escherichia coli* BL21 (DE3)	Expression strain (Novagen)
*Escherichia coli* BL21 Star (DE3)	Expression strain (Novagen)
*Escherichia coli* ML-35p	Laboratory strain (ATCC collection)
*Escherichia coli* C600	Laboratory strain (ATCC collection)
*Escherichia coli* (XDR CI 1057)	Extensively drug resistant clinical isolate (urine, urinary tract infection; ESBL+)
*Escherichia coli* (CI 214)	Clinical isolate (urine, acute pyelonephritis)
*Klebsiella pneumoniae* (CI 287)	Clinical isolate^∗^
*Klebsiella pneumoniae* (XDR CI 1056)	Extensively drug resistant clinical isolate (urine, urinary tract infection; ESBL+)
*Enterobacter cloacae* (XDR CI 4172)	Extensively drug resistant clinical isolate^∗^ (MBL+)
*Acinetobacter baumannii* (XDR CI 2675)	Extensively drug resistant clinical isolate^∗^ (MBL+)
*Pseudomonas aeruginosa* PAO1	Laboratory strain (ATCC collection)
*Pseudomonas aeruginosa* (XDR CI 1049)	Extensively drug resistant clinical isolate (urine, kidney stone disease; MBL+)
*Proteus mirabilis* (XDR CI 3423)	Extensively drug resistant clinical isolate^∗^ (MBL+)

*CI, clinical isolate; ^∗^, no data available on strain source; XDR, extensively drug resistant strain; ESBL+, extended spectrum beta-lactamase producing strain; MBL+, metallo-beta-lactamase producing strain*.

### Expression and Purification of the Antimicrobial Peptides

The recombinant plasmids for expression of the goat cathelicidins were constructed with the use of pET-based vector as described previously (Panteleev and Ovchinnikova, 2017). The target peptides were expressed in *E. coli* BL21 (DE3) as chimeric proteins that included 8 × His tag, the *E. coli* thioredoxin A with the M37L substitution (TrxL), methionine residue, and a mature cathelicidin. The ChMAP-28 amino acid sequence was translated from mRNA for the corresponding precursor protein (GenBank: AJ243126.1) as a 27-residue peptide without the C-terminal glycine, a common amidation signal in cathelicidins. The transformed *E. coli* BL21 (DE3) cells were grown up to OD_600_ 1.0 at 37°C in lysogeny broth (LB) containing 20 mM glucose, 1 mM MgSO_4_, and 0.1 mM CaCl_2_, 100 μg/ml of ampicillin and then were induced with isopropyl β-D-1-thiogalactopyranoside (IPTG) at a final concentration of 0.3 mM. The cells were cultivated for 5 h at 30°C with intense agitation. Then the cells were pelleted by centrifugation and sonicated in immobilized metal affinity chromatography (IMAC) loading buffer containing 6 M guanidine hydrochloride. The clarified lysate was applied on a column packed with Ni Sepharose (GE Healthcare). The recombinant protein was eluted with the buffer containing 0.5 M imidazole. Then the eluate containing the fusion protein was acidified (up to pH 1.0) and cleaved by 100-fold molar excess of cyanogen bromide over methionine for 20 h at 25°C in the dark. The reaction products were lyophilized, dissolved in water, titrated to pH 5.0, and loaded on a semi-preparative Reprosil-pur C18-AQ column (10 mm × 250 mm, 5-μm particle size, Dr. Maisch GmbH). Reversed-phase high-performance liquid chromatography (RP-HPLC) was performed with a linear gradient of acetonitrile in water containing 0.1% trifluoroacetic acid. The peaks were monitored at 214 and 280 nm. The collected fractions were analyzed by MALDI-TOF mass-spectrometry using Reflex III instrument (Bruker Daltonics). The fractions containing the target peptides were lyophilized and dissolved in water. The synthetic melittin (>98% pure) was kindly provided by Dr. Sergey V. Sychev (M.M. Shemyakin and Yu. A. Ovchinnikov Institute of Bioorganic Chemistry of the Russian Academy of Sciences, Moscow, Russia). The recombinant tachyplesin-1 was obtained as described previously (Panteleev and Ovchinnikova, 2017). The peptides concentrations were estimated using UV absorbance.

### Circular Dichroism Spectroscopy and Structure Analysis

Secondary structures of the cathelicidins were analyzed in different environments by circular dichroism spectroscopy (CD) with the use of Jasco J-810 instrument (Jasco) at 25°C. The experiment was performed in 10 mM sodium phosphate buffer (NAPB, pH 7.4), phosphate-buffered saline (PBS, pH 7.4), 50% TFE (Sigma), 30 mM DPC (Anatrace) micelles, and 30 mM SDS (Sigma) micelles. Final concentrations of the peptides were of 300 μM. Four consecutive scans were performed and averaged, followed by subtraction of the blank spectrum of the solvent. The CONTINLL program was used for data analysis (Provencher and Glöckner, 1981). Homology modeling was performed by MODELLER software (Sali and Blundell, 1993). The spatial structures were visualized by Chimera software (Pettersen et al., 2004).

### Hemolysis and Cytotoxicity Assay

Hemolytic activity of the peptides was tested against the fresh suspension of human red blood cells (hRBC) using the hemoglobin release assay as described previously (Panteleev et al., 2016). Three experiments were performed with the hRBC from blood samples obtained from independent donors. The obtained data were represented as average means with standard deviations. The cytotoxicity of the peptides against HEK293T (transformed human embryonic kidney cells) and HEF (human embryonic fibroblasts) cell lines was studied using the colorimetric 3-(4,5-dimethylthiazol-2-yl)-2,5-diphenyltetrazolium bromide (MTT) dye reduction assay according to (Panteleev and Ovchinnikova, 2017). Three independent experiments were performed for each peptide. Half maximal inhibitory concentration (IC_50_) values were estimated as described previously (Kuzmin et al., 2018).

### Antimicrobial Assay

Antimicrobial assay was performed as described previously (Panteleev et al., 2017b). Briefly, mid-log phase bacterial test cultures were diluted with the 2× Mueller-Hinton broth (MH, Sigma) supplemented with 1.8% NaCl or without it so that to reach a final cell concentration of 10^6^ CFU/ml. 50 μl aliquots of the obtained bacterial suspension were added to 50 μl of the peptide solutions serially diluted with 0.1% water solution of bovine serum albumin (BSA) in 96-well flat-bottom polystyrene microplates (Eppendorf #0030730011). After incubation at 37°C and 900 rpm for 24 h the minimum inhibitory concentrations (MIC) were determined as the lowest peptide concentrations that prevented growth of a test microorganism observed as visible turbidity. The results were expressed as the median values determined on the basis of at least three independent experiments performed in triplicate.

### Checkerboard Assay

The peptides were twofold serially diluted with 0.1% BSA in 96-well microplates (Eppendorf #0030730011). Then, the peptide solutions were mixed in the new test plate crosswise in such a way that the resulting checkerboard contained each combination of the cathelicidins in eight doubly increasing concentrations, with wells containing the highest concentration of each peptide at opposite corners (Berditsch et al., 2015). Then, the antimicrobial assay was performed as described in the previous section. MICs were defined as the lowest concentrations of the peptides (when used individually or in the mix with another peptide at a sub-inhibitory concentration) that completely inhibited bacterial growth. The results were expressed as the median values determined on the basis of three independent experiments performed in duplicate. Estimation of synergistic effects of different cathelicidins was performed by calculating the fractional inhibitory concentration index (FICI) according to the equation: FICI = [À]/MIC_À_ + [B]/MIC_B_, where MIC_À_ and MIC_B_ are the MICs of the individual substances, while [A] and [B] are the MICs of A and B when used together. A synergistic effect was defined at a FICI ≤ 0.5.

### Biofilm Assay

Biofilm formation assay was performed as described previously (Panteleev et al., 2017a) with some modifications. Different *E. coli* strains and cultivating conditions were preliminary tested to achieve a strong biofilm formation (Supplementary Figure S2). The *E. coli* CI 214 cells were incubated in the trypticase soy broth (TSB) for 16 h at 37°C and then were diluted 150-fold with the 2×M9 minimal medium supplemented with 50 mM glucose, 10 μM thiamine, 2 mM MgSO_4_, 1 mM CaCl_2_, and the trace metals mixture. 50 μl of the obtained bacterial suspension were added to 50 μl aliquots of the peptide solutions serially diluted with sterilized water in 96-well microplates (Eppendorf #0030730011). The plates were incubated at 32°C with gentle agitation (120 rpm) for 24 h to allow biofilm formation. Then, planktonic (unattached) cells were transferred into the new 96-well plate and OD_620_ of the cell culture was measured with the use of a microplate reader. The wells of the former plate were washed with PBS twice, and the formation of sessile biofilms was analyzed by crystal violet (CV) staining. Briefly, 160 μl of 0.1% crystal violet (CV, Sigma) solution was transferred to each well. The plates were incubated at 25°C for 40 min and rinsed with distilled water to remove an excess of CV. Then the samples were dried for 10 min, and 160 μl of 96% ethanol (v/v) was added to the wells so that to dissolve the CV. 40 min later, the obtained extracts were transferred to a new 96-well plate. The absorption at 570 nm was measured with the use of a microplate reader. The experimental data were obtained from at least three independent experiments performed in triplicate. The results were reported relative to untreated bacteria served as a control. The results were analyzed using the GraphPad Prism 6.0 software.

### Resistance Induction Experiments

Resistance induction experiments were performed using the previously described method (Chernysh et al., 2015) with some modifications. Briefly, on day 1, the overnight culture of wild-type bacteria was diluted with the 2× MH broth containing 1.8% NaCl so that to reach a final cell concentration of 10^6^ CFU/ml. 50 μl aliquots of the obtained bacterial suspension were added to 50 μl of the peptide solutions serially diluted with 0.1% water solution of BSA in 96-well microplates (Eppendorf #0030730011). After incubation at 37°C and 900 rpm for 22 ± 2 h, MICs were determined as described above. For each subsequent daily transfer, 5 μl of the inoculum taken from the first well with a sub-inhibitory drug concentration were diluted with 1 ml of the fresh 2× MH broth supplemented with 1.8% NaCl. Then, 50 μl of this suspension were sub-cultured into the next passage wells containing 50 μl aliquots of the peptide at concentrations from 0.25× to 16× of the current MIC of each agent. 26 repeated passages in the presence of antimicrobial agents were made for each bacterial strain during the experiment. Typically, the experiment was finished when the bacterial culture became resistant to antibiotic polymyxin B (Applichem) used as a control. Finally obtained cell cultures were passaged five times in the absence of antimicrobial agent to confirm that the acquired resistance is stable. Control serial passages in the absence of the agent were also performed. The obtained cultures showed unchanged MICs against antibacterial agents.

### Bacterial Membranes Permeability Assay

To examine an ability of the peptides to affect the barrier function of outer and inner membranes of Gram-negative bacteria, we slightly modified the previously described procedure (Shamova et al., 2016) with the use of the *E. coli* ML-35p strain constitutively expressing cytoplasmic β-galactosidase and lacking lactose permease, and also containing β-lactamase in the periplasmic space. The state of the *E. coli* ML-35p outer and cytoplasmic membranes was assessed based on their permeability to chromogenic markers nitrocefin (Calbiochem-Novabiochem) and *o*-nitrophenyl-β-D-galactopyranoside (ONPG, AppliChem) which are the β-lactamase and β-galactosidase substrates, respectively. The cells were incubated in the TSB medium at 37°C for 16 h, washed three times with 10 mM sodium phosphate buffer (pH 7.4) to remove residual growth media, diluted to the concentration of 2.5 × 10^8^ CFU/ml. The experiments were performed in 10 mM sodium phosphate buffer with or without 0.9% NaCl. The final concentration of *E. coli* ML-35p cells was of 2.5 × 10^7^ CFU/ml. The concentrations of ONPG and nitrocefin were of 2.5 mM and 20 μM, respectively. Peptide samples were placed in the wells of a 96-well plate with non-binding surface (NBS, Corning #3641), and the optical density (OD) of the solution rising due to the appearance of the hydrolyzed nitrocefin or ONPG was measured at 540 and 405 nm, respectively, using the Multiskan EX microplate reader (Thermo Fisher Scientific). The final volume in each well was 200 μl. The experiments were performed at 37°C under stirring at 300 rpm. Control experiments were performed under the same conditions without addition of a peptide. Three independent experiments were performed, and the curve pattern was similar for all three series.

### Flow Cytometry

The *E. coli* ML-35p cells were incubated in the TSB medium for 16 h at 37°C and washed as described above. Bacterial cell suspensions were then incubated for 1 h at 37°C with peptides at different concentrations prepared by twofold serial dilution. The assay was performed in the 96-well NBS microplates in 10 mM sodium phosphate buffer with or without 0.9% NaCl (pH 7.4). Then, SYTOX green (Life Technologies) was added to the treated cells at a final concentration of 2.5 μM and incubated for 10 min at room temperature in the dark. The SYTOX green does not penetrate live cells, but once inside the cell it binds to nucleic acids resulting in more than 500-fold enhancement of fluorescent emission. The fluorescence of the bacterial suspensions diluted 5-fold with PBS was measured (λ_Exc_ = 488 nm, λ_Em_ = 530 nm) by NovoCyte flow cytometer (ACEA Biosciences). For each sample 10^5^ events were recorded. Fluorescence signals were expressed as a percentage of two distinct cell groups: (1) healthy and partially damaged cells were deemed as totaling from 10^2^ to 10^5^ range of detection at 530 nm; (2) completely permeabilized (dead) cells were deemed as amounting ≥10^5^ range of detection at 530 nm. Two independent experiments were performed, and the similar results were obtained.

### Cell-Free Protein Expression Assay

The cell lysate used for translation inhibition assay was prepared using the *E. coli* BL21 Star (DE3) cell culture grown at 30°C in the 2x YTPG liquid medium (1% yeast extract, 1.6% tryptone, 0.5% NaCl, 22 mM NaH_2_PO_4_, 40 mM Na_2_HPO_4_, 0.1 M glucose). The chromosome of DE3 strains contains a gene encoding T7 RNA polymerase under control of the *lacUV5* promoter. The bacterial culture was grown to OD_600_ 0.8–1.0, then T7 RNA polymerase gene was induced by adding 0.2 mM IPTG. Bacteria were harvested at OD_600_ 5.0–6.0 by centrifugation (3000 *g*, 30 min, 4°C). The bacterial pellet was washed three times by suspending it in four volumes of wash buffer (10 mM Tris-acetate buffer, pH 8.2, 60 mM potassium glutamate, 14 mM magnesium acetate, 1 mM DTT), then resuspended in one volume of the same buffer (1 ml per 1 g of wet cell mass) and disrupted by sonication at 5–15°C. The total cell lysate was centrifuged at 15000 *g* (30 min, 4°C). The supernatant was split into aliquots and stored at -70°C.

In order to investigate the effect of AMPs on the translation process, the peptides were added to a cell-free protein synthesis (CFPS) reaction mix with a plasmid encoding EGFP variant (F64L, S65T, Q80R, F99S, M153T, and V163A) under a control of the T7 promoter. The reaction mix consisted of the following components: 1.2 mM ATP, 0.8 mM UTP, 0.8 mM GTP, 0.8 mM CTP, 2 mM of each of 20 proteinogenic amino acids, 1.5 mM spermidine, 1 mM putrescine dihydrochloride, 0.06647 mM calcium folinate, 170 ng/ml tRNA from the *E. coli* MRE 600 strain, 0.33 mM NAD, 120 mM HEPES-KOH (pH 8.0), 10 mM ammonium glutamate, 175 mM potassium glutamate, 60 mM glucose, 15 mM magnesium glutamate, 2% PEG 8000, 25% *E. coli* BL21 Star (DE3) cell lysate, 10 ng/μl plasmid DNA. The reaction volume was 50 μl. The peptides were dissolved in PBS with the addition of 0.1% BSA. Streptomycin and erythromycin were used in the positive control reactions. Fluorescence of the sample without inhibitor was set as the 100% value. The reaction proceeded for 1.5 h in 96-well clear flat-bottom black polystyrene microplates (Corning #3340) sealed with Parafilm in a plate shaker (30°C, 900 rpm). Fluorescence of the synthesized EGFP was measured with the microplate reader AF2200 (λ_Exc_ = 488 nm, λ_Em_ = 510 nm). The experimental data were obtained from at least three independent experiments. IC_50_ values were determined by interpolation from non-linear regression curves using the GraphPad Prism 6.0 software.

### Electrophoretic Mobility Shift Assay

The peptides binding to DNA was examined by electrophoretic mobility shift assay (EMSA) according to the previously described protocol (Panteleev et al., 2016). Briefly, the plasmid pUC19 was incubated with the tested peptides at increasing concentrations in the binding buffer containing 10 mM Tris-HCl (pH 8.0), 50 μg/ml BSA, 5% glycerol, 1 mM DTT, 150 mM NaCl, 20 mM KCl, and 1 mM EDTA, at 25°C for 30 min. Then, the samples were analyzed by electrophoresis in 0.8% agarose gel. The DNA migration was detected by means of the ethidium bromide fluorescence tracking. The DNA-peptide (w/w) ratios were of 1:0 (negative control), 4:1, 2:1, 1:1, 1:2, respectively.

### Genetic Analysis of Bacterial Strains

The *sbmA* and *yaiW* genes encoding the *E. coli* inner or outer membrane proteins, respectively, as well as a regulatory part of their common operon were amplified by polymerase chain reaction (PCR) using gene-specific primers (Supplementary Figure S3). Individual bacterial colonies of the tested strain were picked up from Petri dish and used as a template for PCR. The following components were mixed for the PCR: 2 μl of 10× Encyclo buffer (Evrogen), 0.4 μl of 50× Encyclo DNA polymerase (Evrogen), 10 μM forward primer, 10 μM reverse primer, 0.2 mM dNTPs, bacterial cells on inoculation loop, and water diluting to the total volume of 20 μl. Amplification was carried out on a thermocycler using: initial denaturation (95°C, 10 min), 25 amplification cycles (94°C, 30 s; 55°C, 40 s; 72°C, 90 s), and final elongation (72°C, 10 min). The products were separated by electrophoresis on 1.5% agarose gel (4 V/cm) and visualized on a UV trans-illuminator. The PCR products were purified from agarose gel and inserted into pGEM-T vector (Promega). The ligation products were transformed into the chemically competent *E. coli* DH10B cells. Plasmid DNA was isolated from overnight cultures of single white colonies on LB agar plates supplemented with ampicillin (100 μg/ml), using Plasmid Miniprep kit (Evrogen). The plasmids were sequenced on both strands using the ABI PRISM 3100-Avant automatic sequencer (Applied Biosystems). At least two independent experiments were performed with each strain to prove the obtained results.

## Results

### Expression and Purification of the Recombinant Peptides

Natural goat cathelicidins do not undergo significant post-translational modifications, therefore heterologous expression in *E. coli* of the peptides fused with a carrier protein seems to be a reasonable approach for their production. The goat cathelicidins were produced using the same protocol. To facilitate the purification process and improve final yield, the recombinant peptides were obtained as fusion proteins with the N-terminal 8×His tag and thioredoxin A which was approved to be an effective carrier protein for different peptide scaffolds having antibacterial activity (Li, 2011). The peptides were purified by a downstream process including IMAC of the clarified total cell lysate, cleavage of the fusion protein with cyanogen bromide, and fine purification by RP-HPLC (Supplementary Figure S1). Final yields of ChMAP-28, mini-ChBac7.5Nα, and mini-ChBac7.5Nα(1–16) were 3.4, 9.2, and 7.5 mg per 1 l of the culture medium, respectively. The obtained recombinant cathelicidins were analyzed by MALDI-TOF mass-spectrometry. The measured m/z values of the cathelicidins matched the corresponding calculated molecular masses (Supplementary Table S1).

### Secondary Structure of Goat Cathelicidins

In this study, CD spectroscopy was used to analyze the secondary structure of the goat cathelicidin ChMAP-28. As shown in Figure 1A, the CD spectra of ChMAP-28 dissolved in phosphate buffer or phosphate-buffered saline showed a negative peak at the wavelength of 200 nm, which indicated that it mainly adopted random coil conformation. Therefore, the above conditions do not facilitate the peptide folding. In contrast, the CD spectra of ChMAP-28 interacted with SDS or DPC micelles showed a strong positive peak at 195 nm, and two negative peaks at 208 and 220 nm, which indicated that ChMAP-28 mainly adopted α-helix secondary structures in hydrophobic environments. Indeed, the peptide has a relatively high homology to known α-helical cathelicidins from *Bos taurus*: 61% sequence identity with BMAP-27 and 42% – with BMAP-28 (Figure 1B). We performed homology modeling based on the BMAP-27 structure to visualize a probable spatial structure of ChMAP-28 in membrane-mimicking environment (Figures 1C,D). Significant homology between mini-ChBac7.5Nα and the N-terminal fragment of Bac7 suggests their similar structure, thus mini-ChBac7.5Nα was not analyzed by CD-spectroscopy. It is assumed that mini-ChBac7.5Nα, alike the peptide Bac7(1–16), adopts extended structures within the bacterial ribosomal exit tunnel (Figure 1E). Generally, mini-ChBac7.5Nα and its orthologs from mammalian species (artiodactyls and cetaceans) recorded in the Genbank showed a relatively high homology, especially between sequences at the N-terminal and central parts of the peptides (Figure 1F). Interestingly, recent studies revealed that the Bac7 homolog, isolated from the bottlenose dolphin *Tursiops truncatus* and designated as Tur1B, was enriched with Trp residues and displayed rather modest inhibitory effect on bacterial translation (Mardirossian et al., 2018).

### Cytotoxic Properties of Goat Cathelicidins

**FIGURE 2:**
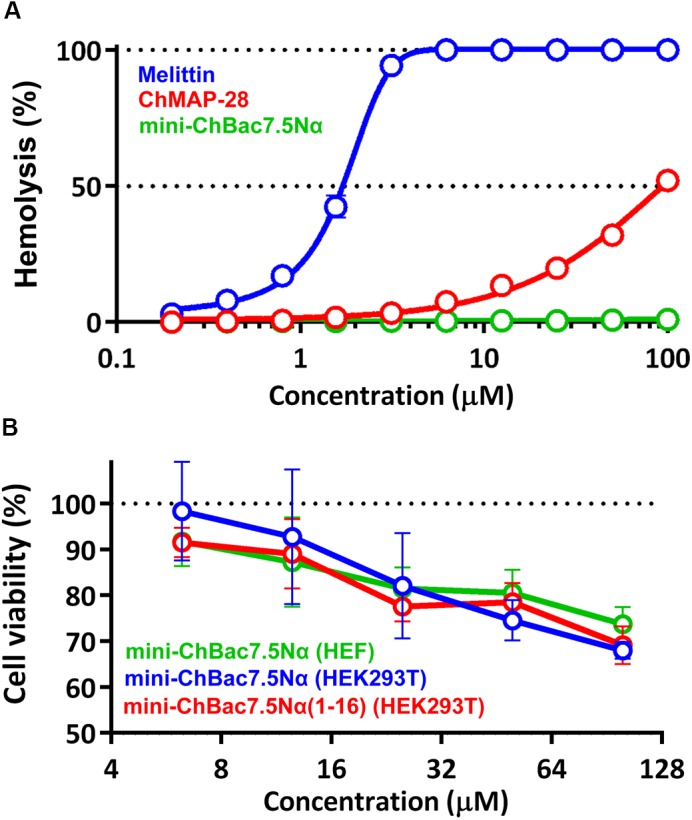
**(A)** Hemolytic activity of the goat cathelicidins and melittin after 1.5 h incubation (hemoglobin release assay). **(B)** Cytotoxicity of mini-ChBac7.5Nα and its shortened analog toward HEK293T (transformed human embryonic kidney cells) and HEF (human embryonic fibroblasts) cells after 24 h incubation (MTT-assay). Three independent experiments were performed with each peptide.

To estimate cytotoxic effect of the cathelicidins, human red blood cells (hRBC) as well as adhesive cell lines of human embryonic fibroblasts (HEF) and human embryonic kidney cells (HEK293T) were used. Melittin known as a potent cytolytic peptide was used as a positive control. It is known that most Pro-rich AMPs have no pronounced toxicity to mammalian cells. Earlier, we showed that cytotoxicity of mini-bactenecins at concentrations up to 30 μM against a set of mammalian cell lines after 24 h was quite modest (Shamova et al., 2016). However, the peptides are not completely non-toxic. The data analysis revealed that both mini-ChBac7.5Nα and its shortened analog showed cytotoxic activity against mammalian cell lines at concentrations >25 μM (Figure 2). Mini-ChBac7.5Nα almost lacked hemolytic activity and lysed only 2% of red blood cells at the concentration of 100 μM. In contrast, a half maximal hemolysis concentration (HC_50_) of ChMAP-28 was of ∼100 μM, and the peptide had the IC_50_ against HEK293T cells of ∼3.5 μM. Interestingly, its bovine ortholog BMAP-28 possessed the IC_50_ against murine 3T3 cells and HC_50_ of <3.75 and ∼20 μM, respectively (Ahmad et al., 2009). Melittin was proved to be significantly more toxic than α-helical cathelicidins and completely damaged all the cells tested at concentrations of <2.5 μM.

### Antimicrobial Activity of Goat Cathelicidins

Amphiphilic AMPs are known to be adsorbed on plastic surfaces (Wiegand et al., 2008). For these reason, serial dilutions of the peptides were performed in the presence of BSA in the growth medium in order to minimize this effect. MICs of goat cathelicidins and melittin against Gram-positive and Gram-negative bacteria are presented in Table 2. It was reported that Pro-rich AMPs have high antimicrobial activity against Gram-negative bacteria and are less active or inactive against most Gram-positive bacteria. In whole, our results confirmed this. It is noteworthy that the medium formulation as well markedly affects the activity values of Pro-rich AMPs. Antibacterial activities of some insect Pro-rich AMPs was low when tested in the presence of a salt, which might inhibit absorption of the peptides to the bacterial surface (Gennaro et al., 2002). Therefore, the salt influence on the antibacterial activity was investigated in this study. Indeed, the presence of 0.9% NaCl resulted in several-fold decrease in the activity of mini-bactenecins against all the strains tested. The shortened analog mini-ChBac7.5Nα(1–16) was shown to be less active and more salt-sensitive as compared with the wild-type mini-ChBac7.5Nα. Interestingly, antibacterial activities of the peptides were similar when tested in a salt-free medium against Gram-negative bacteria *E. coli, Acinetobacter baumannii, Klebsiella pneumoniae*, and *Enterobacter cloacae*. In contrast to the other tested strains, these bacteria have the ABC transport system based on the homodimeric cytoplasmic membrane protein SbmA. In *E. coli* the cytoplasmic membrane protein SbmA and outer membrane lipoprotein YaiW participate in transport of some Pro-rich AMPs and bacteriocins (Arnold et al., 2014). Mutation or deletion of either SbmA or YaiW significantly decreased the ability of the Bac7 to internalize, and significantly reduced susceptibility to the peptide (Arnold et al., 2014). Our results indicated that the presence of the C-terminal fragment PRPRPR did not influence the efficiency of the peptide translocation via SbmA transporter in a salt-free medium, but could play a key role when acting against SbmA-deficient bacteria (e.g., Gram-positive bacteria) or applying in the presence of a salt. Previous study of the Pro-rich pig cathelicidin PR-39 revealed that an activity of the full length peptide was hardly affected by 100 mM NaCl while the shortened peptide derivatives lacked most of their antimicrobial properties under the same conditions (Veldhuizen et al., 2014). It is likely that the observed effect occurs due to electrostatic interactions between positively charged peptides and negatively charged bacterial membranes. Antimicrobial activity of mini-bactenecins seems to be a function of a total charge of the peptide rather than of a charge density and overall hydrophobicity, since mini-ChBac7.5Nα(1–16) has both higher charge-to-length ratio and longer retention time in reversed-phase HPLC (Supplementary Figure S1) than the wild-type mini-ChBac7.5Nα. A total charge could be important at initial stages of Pro-rich AMPs interaction with bacteria, i.e., during the primary electrostatic attraction followed by displacement of divalent cations cross-bridging LPS on the cell surface, that destabilized the membrane and led to the peptide self-promoted uptake.

**Table 2:** Antibacterial activity of goat cathelicidins and melittin.

Strain	*sbmA/yaiW^∗∗^*	Minimum inhibitory concentration (μM)^∗^							
		Melittin		ChMAP-28		mini-ChBac7.5Nα		mini-ChBac7.5Nα(1–16)	
		Without NaCl	0.9% NaCl	Without NaCl	0.9% NaCl	Without NaCl	0.9% NaCl	Without NaCl	0.9% NaCl
*M. luteus* B-1314	-/-	0.25	0.5	0.25	0.5	0.125	0.5	1	8
*B. subtilis* B-886	-/-	0.5	0.5	0.25	1	0.25	4	1	>32
*E. faecalis* ATCC 29212	-/-	1	1	4	>8	>32	>32	>32	>32
*S. aureus* ATCC 29213	-/-	1	1	1	2	8	>32	>32	>32
*S. aureus* 209P	-/-	2	16	0.06	0.5	2	16	8	>32
*E. coli* C600	+/+	4	8	0.06	0.125	2	4	2	16
*E. coli* ML-35p	+/+	2	8	0.06	0.06	0.5	4	0.5	8
*P. aeruginosa* PAO1	-/-	4	8	0.25	1	2	>32	16	>32
*A. baumannii* (XDR CI 2675)	+/-	2	8	0.03	0.25	2	>32	4	>32
*K. pneumoniae* (CI 287)	+/+	4	16	0.125	0.5	4	16	4	>32
*E. cloacae* (XDR CI 4172)	+/+	2	8	0.125	0.25	1	4	1	>32

*^∗^Antibacterial testing was performed in the Mueller-Hinton broth at 37°C.****^∗∗^****The presence of genes encoding the membrane transporters SbmA and YaiW that affect sensitivity to proline-rich antimicrobial peptides (Arnold et al., 2014).*

Cathelicidin ChMAP-28 exhibited significantly more potent antibacterial activity (≥16-fold higher) than melittin against most strains tested. ChMAP-28 was shown to be less sensitive to high ionic strength as compared with mini-bactenecins. ChMAP-28 and last line antibiotics polymyxin B and meropenem were tested against extensively drug resistant clinical isolates of Gram-negative bacteria which belong to “ESKAPE” pathogens: *E. coli, K. pneumoniae, A. baumannii, P. aeruginosa, E. cloacae, P. mirabilis* (Supplementary Table S2). Generally, ChMAP-28 exhibited a potent antimicrobial activity comparable with that or even higher than that of the above mentioned control antibiotics. The peptide was shown to effectively kill all the bacteria including polymyxin- and meropenem-resistant strains, thus arguing against cross-resistance to the peptide.

### Synergy Between Goat Cathelicidins

Antimicrobial activity of most Pro-rich AMPs including mini-bactenecins is reduced at physiological salt concentrations. In view of this, interaction with other co-localized membrane-active molecules may enhance or restore the activity of Pro-rich AMPs. To check the assumption, antibacterial effects of the combination of the goat cathelicidins mini-ChBac7.5Nα and ChMAP-28 were evaluated in the medium containing a physiological concentration of NaCl (Table 3). A set of Gram-negative bacterial species and one Gram-positive strain *S. aureus* 209P sensitive to mini-bactenecins were used as the test microorganisms. To reduce adsorption of AMPs on plastic surfaces while testing antimicrobial activity *in vitro*, we used 0.1% BSA for serial dilutions (Wiegand et al., 2008; Bolosov et al., 2017). In combination with ChMAP-28 at sub-inhibitory concentrations, mini-ChBac7.5Nα exhibited antimicrobial activity with more than fourfold decreased MIC values that led to FICI values of ≤0.375 against different *E. coli* strains. The peptides showed a strong synergistic effect against *K. pneumoniae, E. cloacae*, *A. baumannii* with at least an eightfold decrease in MICs for both agents and FICI values of 0.25, 0.25, and 0.133, respectively. Interestingly, the presence of ChMAP-28 either completely restored or slightly increased the activity of mini-ChBac7.5Nα as compared with that evaluated in a salt-free medium against these bacterial strains (Table 2), including the clinical isolate of *E. coli* CI 214 with a weak sensitivity to mini-ChBac7.5Nα (the MIC values were of 4 μM in a salt-free medium and >64 μM in the presence of 0.9% NaCl). As described above, all the mentioned strains normally have the SbmA transport system. It suggests that ChMAP-28 acting at sub-inhibitory concentrations may promote translocation of mini-ChBac7.5Nα through the outer membrane, which is an obstacle to Pro-rich AMPs when electrostatic interactions are affected by increased ionic strength. Inside the periplasmic space mini-ChBac7.5Nα can effectively use cytoplasmic membrane transporters to get into the cell. At the same time, ChMAP-28 did not restore the activity of mini-ChBac7.5Nα against *P. aeruginosa*, and no synergy was observed. These findings are consistent with the previous study that revealed the lack of synergy between Pro-rich AMPs and the membranolytic peptide CRAMP while testing antimicrobial activity against *P. aeruginosa* (Knappe et al., 2016). Surprisingly, a pronounced synergistic effect was observed against *S. aureus* 209P with MICs of both peptides almost identical to those measured in a salt-free medium, thus resulting in FICI of 0.188. This observation allowed us to speculate that some Gram-positive bacterial strains might have transport systems for Pro-rich AMPs. On the other hand, the presence of Pro-rich AMPs could interact with the structures of cell wall teichoic acids, the anionic glycopolymers, and thereby helped ChMAP-28 molecules to reach lipid bilayer.

**Table 3:** Synergy between goat cathelicidins ChMAP-28 and mini-ChBac7.5Nα.

Strain	ChMAP-28			mini-ChBac7.5Nα			FICI^∗^	Synergy
	MIC_A_	[A]	FIC_A_	MIC_B_	[B]	FIC_B_		
*E. coli* BL21 (DE3)	0.06	0.015	0.25	8	1	0.125	0.375	Yes
*E. coli* ML-35p	0.06	0.008	0.125	4	1	0.25	0.375	Yes
*E. coli* C600	0.125	0.015-0.03	0.125-0.25	4	1	0.25	0.375-0.5	Yes
*E. coli* (XDR CI 1057)	0.125	0.008	0.063	8	2	0.25	0.313	Yes
*E. coli* (CI 214)	0.06	0.015	0.25	>64	4	0.031	0.281	Yes
*E. cloacae* (XDR CI 4172)	0.25	0.03	0.125	4	0.5	0.125	0.25	Yes
*K. pneumoniae* (CI 287)	0.5	0.06	0.125	16	2	0.125	0.25	Yes
*A. baumannii* (XDR CI 2675)	0.25	0.03	0.125	>32	0.5	0.008	0.133	Yes
*P. aeruginosa* PAO1	0.125	0.06	0.5	>32	8	0.125	0.625	No
*S. aureus* 209P	0.5	0.03	0.063	16	2	0.125	0.188	Yes

*^∗^The estimation of synergistic effects between cathelicidins was performed by calculating the fractional inhibitory concentration index (FICI) according to the equation: FICI = FIC_*A*_ + FIC_*B*_ = [À]/MIC_*À*_ + [B]/MIC_*B*_, where MIC_*À*_ and MIC_*B*_ are the MICs of individual peptides, while [A] and [B] are the MICs of A and B when used together. A synergistic effect was defined at a FICI ≤ 0.5*.

### Analysis of Membrane-Permeabilizing Activity

**FIGURE 3:**
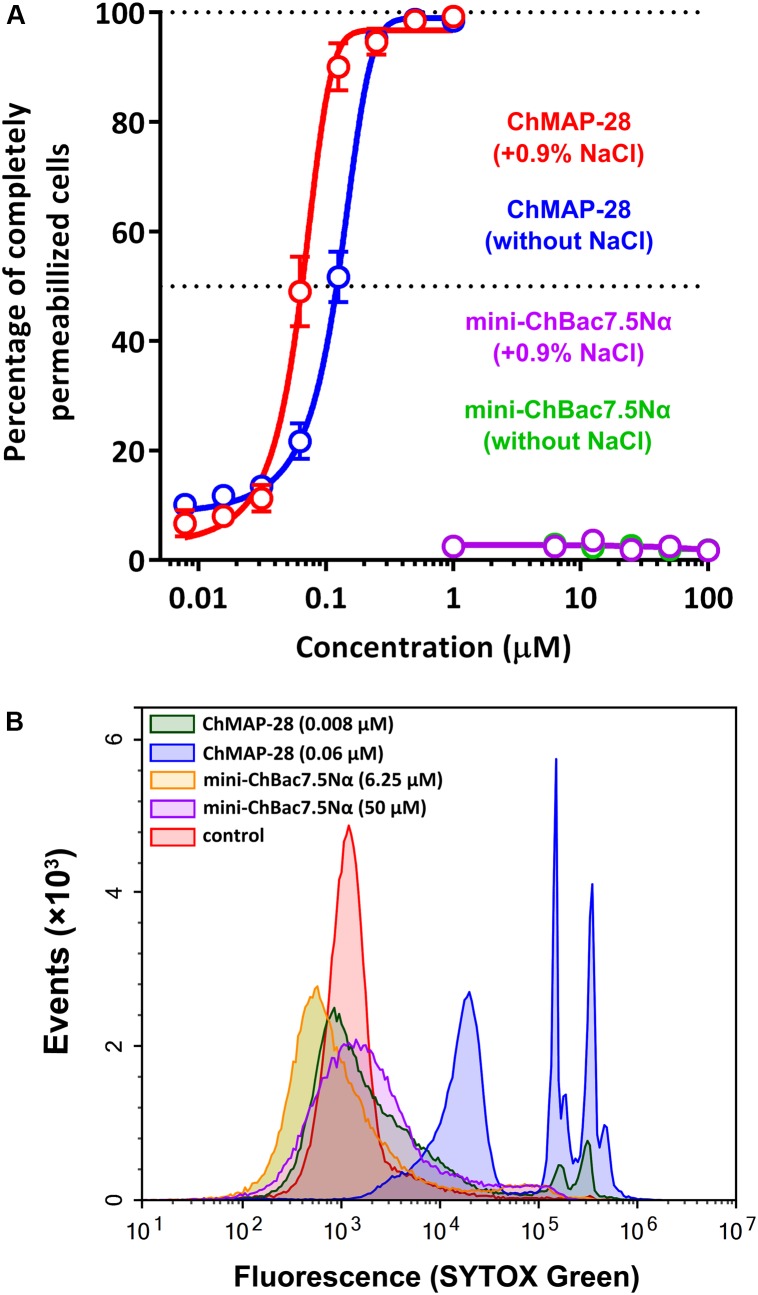
Flow cytometry analysis of the SYTOX Green uptake in *E. coli* ML-35p cells after 60 min treatment with goat cathelicidins. **(A)** The graph showing the effect of the peptides at different concentrations. The experiment was performed in duplicate, with the plotted points representing the mean value ± SD. **(B)** Analysis of bacterial cell populations after treatment in the presence of 0.9% NaCl.

Antimicrobial peptides can realize their biological functions by damaging membrane integrity and specifically inhibiting intracellular processes. One of the most important objectives in functional study of AMPs is to elucidate a mechanism of their antimicrobial action. The effect of the goat cathelicidins on *E. coli* ML-35p membrane integrity was characterized by monitoring both the SYTOX Green uptake by flow cytometry and permeability to chromogenic markers – ONPG and nitrocefin. The membranolytic peptide melittin was used as a positive control. The flow cytometry data show that mini-ChBac7.5Nα does not influence the *E. coli* cytoplasmic membrane integrity regardless of salt concentration (Figure 3A). This is in agreement with our previous data (Shamova et al., 2016). In contrast to the longer peptide Bac7(1–35) (Podda et al., 2006), mini-ChBac7.5Nα did not significantly damage membranes at higher concentrations than the MIC values. The cathelicidin ChMAP-28 was shown to damage bacterial membrane at nanomolar concentrations that led to the appearance of bacterial subpopulations with increased fluorescence intensity by one or two orders of magnitude vs. a control (Figure 3B). These shifted peaks on the graph may represent cells with qualitatively different grades of membrane damage.

**FIGURE 4:**
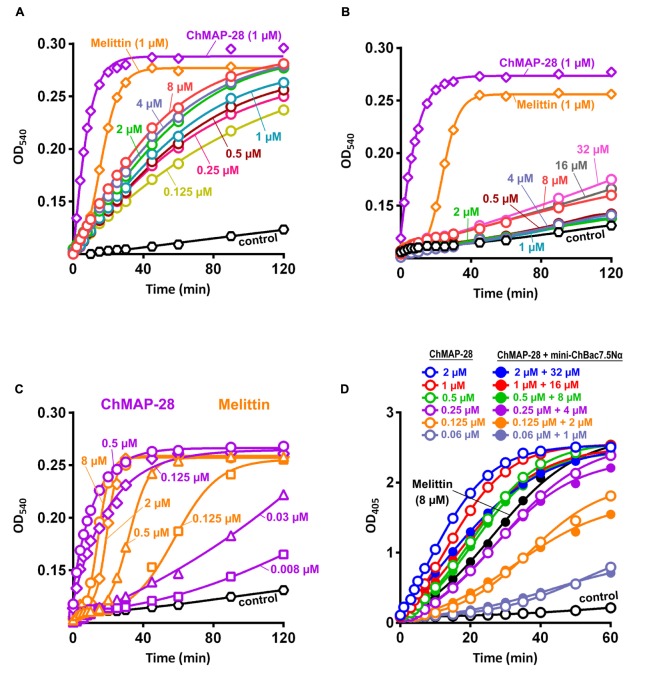
Kinetics of changes in *E. coli* ML-35p outer and cytoplasmic membrane permeability measured with the use of chromogenic markers – the products of nitrocefin (OD_540_) and ONPG (OD_405_) hydrolysis, respectively. Outer membrane permeability resulting from incubation of bacteria with mini-ChBac7.5Nα at various concentrations (from 0.125 to 32 μM, highlighted with colors) in the absence **(A)** or in the presence **(B)** of 0.9% NaCl. Melittin and ChMAP-28 at concentration of 1 μM were used as positive control samples. **(C)** Analysis of outer membrane permeability resulting from incubation with ChMAP-28 or melittin in the presence of 0.9% NaCl. **(D)** Comparative analysis of cytoplasmic membrane permeability resulting from incubation with the individual ChMAP-28 or with its combinations with mini-ChBac7.5Nα. Melittin at concentration of 8 μM was used as a positive control. Three independent experiments were performed, and the curve pattern was similar for the three series.

Synergy between two different AMPs could result from either facilitation of translocation of one of them into the cell by another peptide or cooperative augmentation of the membrane damage, as was shown for cathelicidins and defensins (Nagaoka et al., 2000). To decide between these scenarios, a comparative analysis of the ability of the cathelicidins to disrupt the integrity of inner and outer bacterial membranes was conducted in a wide range of concentrations. Interestingly, mini-ChBac7.5Nα was shown to effectively damage outer membrane in a salt-free environment (Figure 4A). However, the addition of 0.9% NaCl reduced the activity to a modest effect at 8–32 μM (Figure 4B), that could explain a weak antibacterial activity of mini-bactenecins in the presence of salt (Table 2). Translocation of mini-ChBac7.5Nα into periplasmic space likely depends on ability to disrupt the outer membrane, which becomes an impassable barrier in the presence of NaCl. At the same time, ChMAP-28 was proved to damage the outer membrane in a salt-containing medium at concentration of 0.008 μM (Figure 4C) that was equal to the fractional MIC of the synergy combination with mini-ChBac7.5Nα (see Table 3). The data presented in Figure 4D allowed us to rule out the effect of potentiating the cytoplasmic membrane permeabilization: in most cases the presence of mini-ChBac7.5Nα did not significantly affect or even decreased the ability of ChMAP-28 to damage the membrane. The same was true when we tested the peptide mixtures on the *E. coli* ML-35p outer membrane (graphical data not shown). Taken together, these results suggest that ChMAP-28 at sub-inhibitory concentrations promotes translocation of mini-ChBac7.5Nα into the periplasmic space rather than enhances its membrane activity.

### Inhibition of *in vitro* Protein Synthesis in *E. coli*

**FIGURE 5:**
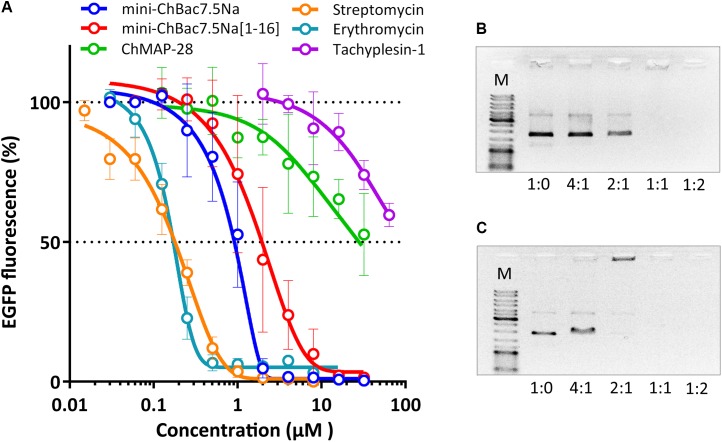
Effects of goat cathelicidins, tachyplesin-1, and conventional antibiotics at different concentrations on the fluorescence resulting from the *in vitro* translation of EGFP with the use of *E. coli* BL21 (DE3) Star cell extract **(A)**. Data are the mean ± SD of at least three independent experiments performed in triplicate. ChMAP-28 **(B)** and mini-ChBac7.5Nα **(C)** binding to DNA was examined by electrophoretic mobility shift assay (EMSA). Various amounts of the peptides were incubated with 100 ng of the pUC19 plasmid DNA, and DNA binding was assessed by the peptide influence on the electrophoretic mobility of DNA. DNA-to-peptide weight ratios are indicated on the horizontal axis. Lane M shows the DNA molecular size marker (500–10,000 bp).

Taking into account the reported data on the mechanism of action of proline-rich AMPs and the inability of mini-ChBac7.5Nα to disrupt cytoplasmic membrane integrity, we tested an ability of this peptide and other antimicrobial compounds to inhibit protein biosynthesis *in vitro*. The experiment was carried out using the bacterial cell-free protein synthesis system expressing the green fluorescent protein (GFP). The results obtained for streptomycin with IC_50_ value of 0.2 μM and a full inhibition of >1 μM correspond with the published data (Krizsan et al., 2014) (Figure 5A). IC_50_ for mini-ChBac7.5Nα was of ∼1 μM which is comparable to that of conventional inhibitors of bacterial translation – streptomycin and erythromycin. Apart from that, the values of IC_50_ for mini-ChBac7.5Nα were similar to those of its homologs – the Bac7 fragments (Seefeldt et al., 2016), and also to the previously determined MICs against *E. coli* (see Table 2). It should be noted that the mini-ChBac7.5Nα(1–16) fragment inhibits biosynthesis twice less effectively than the wild-type mini-ChBac7.5Nα that might account for the reduction of antibacterial activity. Interestingly, the cathelicidin ChMAP-28 also affects protein biosynthesis, but at much higher concentrations than its MIC. It seems that ChMAP-28 ability to inhibit translation is due to a non-specific interaction with nucleic acids. This assumption is supported by the fact that tachyplesin-1, which is known to bind DNA (Yonezawa et al., 1992), demonstrates a comparable level of inhibition. For several cationic AMPs, e.g., for indolicidin (bovine tryptophan-rich cathelicidin), binding to DNA is considered to be one of the mechanisms of their antimicrobial action. AMP-DNA binding induces aggregation and interferes with the process of replication (Hsu et al., 2005). It was shown that both goat cathelicidins bound plasmid DNA at a mass ratio of 1:1 (Figures 5B,C). In addition, ribosome-binding is supposed to be the main factor responsible for bacterial growth inhibition by the *Bos taurus* cathelicidin Bac7. Comparing our results with known data on Bac7 and bearing in mind a high homology degree between mini-ChBac7.5Nα and the N-terminal fragment of Bac7, we assume that the main target for mini-ChBac7.5Nα is also the 70S ribosome. Data obtained allow us to conclude that two goat cathelicidins – ChMAP-28 and mini-ChBac7.5Nα possess essentially different mechanisms of antimicrobial action: ChMAP-28 preferentially acts by increasing cytoplasmic membrane permeability, while mini-ChBac7.5Nα specifically inhibits bacterial translation.

### Anti-biofilm Activity of Goat Cathelicidins

**FIGURE 6:**
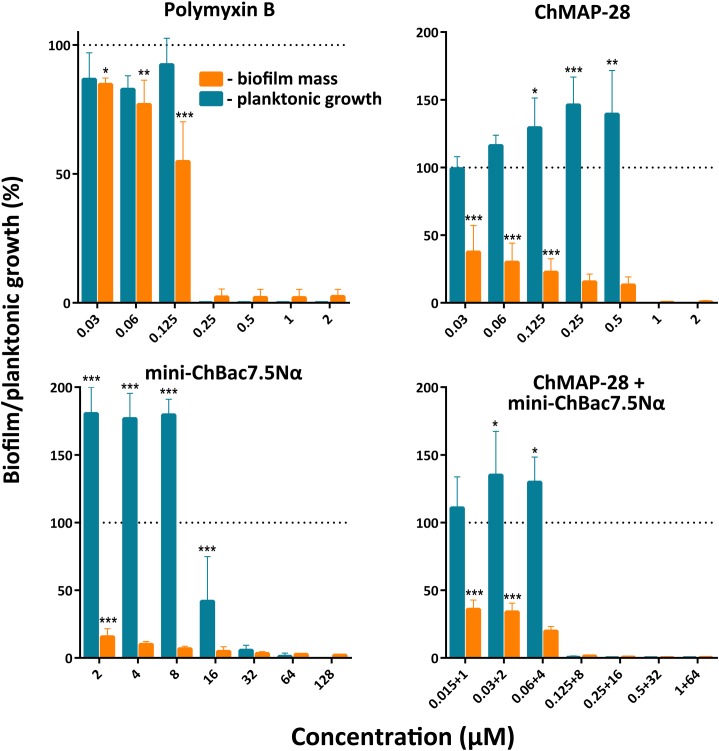
Effect of individual goat cathelicidins ChMAP-28 or mini-ChBac7.5Nα, their synergistic combination, and polymyxin B at different concentrations including sub-inhibitory MICs on planktonic cell growth and biofilm formation of *E. coli* clinical isolate. Biofilm formation was assessed by the colorimetric crystal violet-based technique. The results are expressed as percentage of the planktonic growth or the formed biofilm by reference to an untreated control taken as 100%. Data are the mean ± SD of at least three independent experiments performed in triplicate. ^∗^*P* < 0.05, ^∗∗^*P* < 0.01, ^∗∗∗^*P* < 0.001 significantly different compared to the control.

The biofilm formation raises difficulties for therapy of bacterial infectious diseases due to the resistance to conventional antibiotics. Notably, the biofilms can colonize abiotic objects such as surfaces of medical devices and instruments and also be localized in host-organism tissues. Development of compounds that could prevent adhesion of microorganisms to the surfaces and therefore block the formation of biofilms is one of the key problems of modern medicine. In the present work, we investigated whether the synergistic combination of different goat cathelicidins prevent formation of biofilms. The strain *E. coli* CI 214 isolated from urine in acute pyelonephritis was proved to be a strong biofilm producer when cultivated in minimal growth medium (Supplementary Figure S2). It is important to notice that this strain has comparatively low sensitivity to mini-ChBac7.5Nα (Table 2). All the compounds demonstrated high activity, and at concentrations suppressing planktonic bacterial growth (MIC) the biofilm formation was not observed (Figure 6). Complete inhibition of both planktonic and biofilm growth by ChMAP-28, mini-ChBac7.5Nα, and their combination was achieved at concentrations of 1, 32, and (0.125 + 8) μM, respectively. Therefore, the synergy effect consisting in the complete inhibition of *E. coli* was shown with the FICI value of 0.375. The MIC values shown by antibiotic polymyxin B agreed well with those reported earlier when tested against *P. aeruginosa* PAO1 (Panteleev et al., 2017a). It is noteworthy that reduction of biofilm formation with sub-inhibitory concentrations of mini-ChBac7.5Nα was followed by a significant stimulation (1.5–2-fold) of planktonic growth as compared with a control. The effect of ChMAP-28 and the combination of the peptides was less pronounced. At concentrations up to 1/16× MIC the peptides inhibited biofilm growth by more than twofold. Presumably, the peptides could prevent an adhesion of bacteria to the plate surface.

### Development of Resistance to Goat Cathelicidins

**FIGURE 7:**
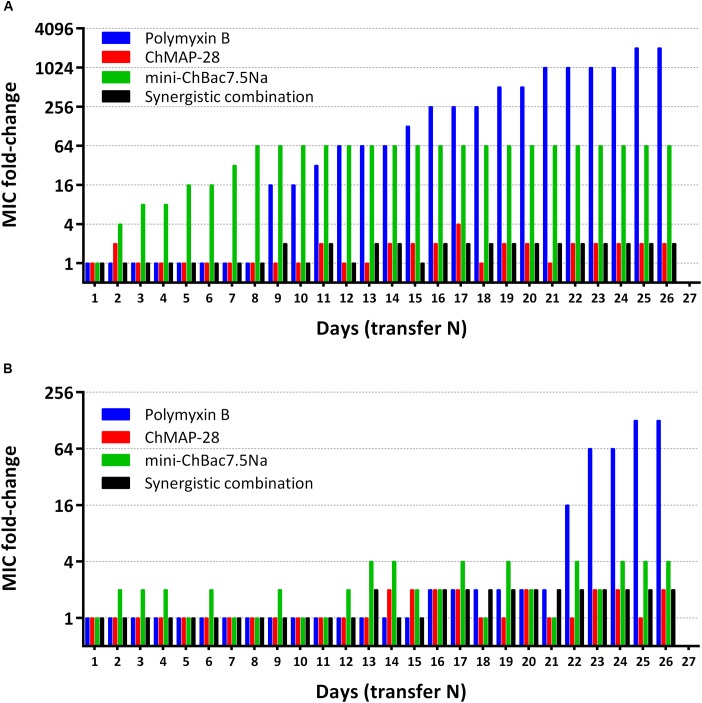
Minimum inhibitory concentrations (MIC) changes in bacterial strains *E. coli* XDR CI 1057 **(A)** and *E. coli* ML-35p **(B)** exposed to selection by individual goat cathelicidins ChMAP-28 (MIC value at transfer “1” = 0.125 μM) or mini-ChBac7.5Nα (MIC value at transfer “1” = 8 μM), the synergistic combination of ChMAP-28 + mini-ChBac7.5Nα (MIC value at transfer “1” = 0.03 + 2 μM, respectively), and the reference antibiotic polymyxin B (MIC value at transfer “1” = 0.125 μM). The experiment was performed in the Mueller-Hinton broth supplemented with 0.9% NaCl at 37°C. 26 repeated passages (transfer N) in the presence of antimicrobial agents were made for each bacterial strain during the experiment.

Capacity of the synergistic combination of the goat cathelicidins to prevent bacterial resistance was investigated. Natural combinations of different AMPs from insects, in contrast to individual peptides and small antibiotic molecules, were proved to prevent resistance development in bacteria (Chernysh et al., 2015). Such approach allows using a lower therapeutic dose of AMPs showing synergy with each other. Two *E. coli* strains (XDR CI 1057 and ML-35p) were subjected to the resistance development test by subsequent culturing in the presence of ChMAP-28, mini-ChBac7.5Nα, or the synergistic combination of the peptides, as well as antibiotic polymyxin B at increasing concentrations. The method used in this study allows to monitor MIC values after each transfer. The 2048- and 128-fold increases in MIC values were registered in the bacterial strains XDR CI 1057 and ML-35p, correspondingly, subjected to selection by polymyxin B after 25 passages (Figure 7). The *E. coli* XDR CI 1057 resistance was developed much earlier, resulting in the MIC value of >256 μM. In both cases, an exponential increase of MICs up to 16-fold was observed as the first step of resistance formation. Susceptibility of *E. coli* ML-35p to mini-ChBac7.5Nα decreased only 4-fold over the whole experiment, and no regular MIC changes were observed. Interestingly, 64-fold increases in MIC value (>256 μM) was registered just after eight passages in the bacterial strain XDR CI 1057 subjected to selection by mini-ChBac7.5Nα, and detectable MIC changes became visible after two initial transfers. Considering that the highest peptide concentration in the experiment was of 256 μM, we cannot exclude that actual MIC was beyond this value. The resistance to mini-ChBac7.5Nα was stable, as a serial passage over five steps in the absence of the peptide did not change the MICs. In contrast, the MICs of ChMAP-28 against both strains increased only twofold after 26 passages. The same was true for the mixture of cathelicidins. Then, resistant strains were analyzed for cross-resistance to other agents tested. The MICs of all the tested antimicrobial agents before and after selection are presented in Table 4. No differences in MICs before and after 26 passages without antimicrobial agents were observed. Susceptibility of the strain to mini-ChBac7.5Nα acquired after incubation with the synergy combination was similar to that of the control strains, thus arguing the presence of membrane active component prevented formation of resistance against Pro-rich AMP. This also suggests that any resistance mechanisms to mini-ChBac7.5Nα developed in our experiment were associated with modification of membrane transporter system but not with mutations of intracellular targets. Notably, we did not observe any cross-resistance of the strains incubated in the presence of cathelicidins to antibiotic polymyxin B used as a control. In contrast, a considerable resistance to mini-ChBac7.5Nα was detected in the polymyxin-resistant strain obtained after selection. The resistance to polymyxins in Gram-negative bacteria can be mediated by modifications of LPS structure and cell surface charge (Soon et al., 2011). It is very likely that such modifications may influence the mini-ChBac7.5Nα activity due to its high dependence on electrostatic interactions and a low hydrophobicity of the peptide.

**Table 4:** Antibacterial activity of goat cathelicidins and polymyxin B against *E. coli* strains obtained after selection experiment.

Strain	Minimum inhibitory concentration (μM)		
	Polymyxin B	ChMAP-28	mini-ChBac7.5Nα
*E. coli* XDR CI 1057 (0 days)	0.125	0.125	8
*E. coli* XDR CI 1057 (26 days without antimicrobial agent)	0.125	0.125	8
*E. coli* XDR CI 1057 (26 days with polymyxin B)	>128	0.25	64
*E. coli* XDR CI 1057 (26 days with mini-ChBac7.5Nα)	0.125	0.125	>128
*E. coli* XDR CI 1057 (26 days with synergy combination)	0.06	0.125	8

### Analysis of Mini-ChBac7.5Nα-Resistant Strain Obtained After Selection Experiment

First, we analyzed an influence of NaCl at physiological concentration on antimicrobial activities of the goat cathelicidins against the *E. coli* XDR CI 1057 wild type strain cultivated without an antimicrobial agent and served as a control and against the strain resistant to mini-ChBac7.5Nα. Both mini-ChBac7.5Nα and its analog mini-ChBac7.5Nα(1–16) were predictably inactive against the resistant strain in the presence of 0.9% NaCl. Surprisingly, mini-ChBac7.5Nα completely restored the activity against the resistant strain with the MIC value of 1 μM when tested in the absence of salt, while the activity of mini-ChBac7.5Nα(1–16) was decreased by eightfold as compared with the wild type strain (see Table 5). It is known that the ABC-transporter SbmA is essential for the Pro-rich AMPs uptake and thus is crucial for their activity (Schmidt et al., 2016). Therefore, the SbmA transporter seems to be a resistance factor. Notably, the antibacterial activity of the Bac7 N-terminal fragments were also shown to be decreased by four–eightfold when tested against the SbmA-deficient *E. coli* strain in the MH medium without salt (Mattiuzzo et al., 2007; Guida et al., 2015). The outer membrane lipoprotein YaiW cotranscribed with SbmA was also shown to influence the activity of the Bac7 N-terminal fragments suggesting involvement of this protein in the SbmA-mediated uptake of the peptide (Arnold et al., 2014). To check the functionality of both proteins, analysis of the *sbmA-yaiW* gene regions of *E. coli* strains was performed (Supplementary Figure S3A). PCR analysis revealed that amplicon lengths for both *sbmA* and *yaiW* genes of the mini-ChBac7.5Nα-resistant *E. coli* strain were identical to those of the control strain (Supplementary Figure S3B). This proves the absence of any notable insertions or deletions in the genes. Earlier, a 600 bp insertion was identified in *sbmA* gene of the *E. coli* strain resistant to Pro-rich AMP apidaecin 1b (Schmidt et al., 2016). All the PCR-products were sequenced, and no difference in a regulatory part of *sbmA* operon of the control and resistant strains tested was found (data not shown). Also, there was no significant difference in the amino acid sequence of YaiW lipoprotein of all the *E. coli* strains tested in this study. The only difference was in the signal peptide mutation (V15A) as compared with *E. coli* BL21 strain. It should be noted that this mutation is quite common among other *E. coli* strains presented in Genbank. Analysis of SbmA revealed the single point mutation V102E in the mini-ChBac7.5Nα-resistant strain as compared with the control one (Supplementary Figure S4). The SAR analysis of SbmA demonstrated that the strains bearing the single mutations (V102G, F219G, or E276G) had a null phenotype for SbmA transport functions (Corbalan et al., 2013). In particular, the *E. coli* V102G mutant strain was almost insensitive to the Bac7(1–16) with the MIC of 156 μM. The residues V102 and F219 are likely involved in the homodimer formation (Corbalan et al., 2013). Apparently, the mutation V102E inactivates SbmA in the strain obtained in this study.

**Table 5:** Effect of salt on activity of goat cathelicidins against *E. coli* strain obtained after 26 days selection in the presence of mini-ChBac7.5Nα.

Strain	Minimum inhibitory concentration (μM)					
	ChMAP-28		mini-ChBac7.5Nα		mini-ChBac7.5Nα(1-16)	
	Without salt	With 0.9% NaCl	Without salt	With 0.9% NaCl	Without salt	With 0.9% NaCl
*E. coli* XDR CI 1057 (26 days without antimicrobial agent)	0.125	0.125	1	8	2	16
*E. coli* XDR CI 1057 (26 days with mini-ChBac7.5Nα)	0.125	0.125	1	>256	16	>256

## Discussion

First known Pro-rich AMPs (apidaecins, bactenecins) were identified 30 years ago in insects and mammals, respectively (Casteels et al., 1989; Gennaro et al., 1989). Mechanism of a typical Pro-rich AMP action against Gram-negative bacteria is accomplished via several steps: (1) electrostatic interaction between negatively charged components of the outer membrane a positively charged peptide; (2) crossing of the outer membrane and getting into the periplasmic space by self-promoted uptake or the membrane damage; (3) translocation by the transporter proteins into the cytosol; (4) interaction with the 70S ribosome. Being the C-terminal part of a large carrier protein, apidaecins were proved to retain the ability to effectively inhibit the growth of bacterial cells during heterologous expression in *E. coli* (Taguchi et al., 1994). Unlike in apidaecins, it is the N-terminus that important for manifestation of the activity of mammalian Bac7-related peptides whereas the C-terminus appears to be variable and less significant (Graf et al., 2017). Therefore, in the present work the Pro-rich mini-bactenecins were expressed as a C-terminal part of the modified thioredoxin A so that to block the active N-terminus. Here, we showed that the protein biosynthesis inhibition is a predominate mechanism of the *Capra hircus* mini-bactenecins action. The membrane activity of the peptides consists in a salt-dependent effect on the outer membrane of Gram-negative bacteria. It should be noted that goat Pro-rich cathelicidins are not completely devoid of toxicity toward mammalian cells. Minor hemolytic activity implies the absence of membranolytic effect on mammalian membranes. However, a linear increase of cytotoxicity toward both HEF and HEK293T cell lines at concentrations up to 100 μM suggests a non-lytic penetration into the cell followed by interaction with an intracellular target. Indeed, the bovine Bac7(1–35) was proved to inhibit eukaryotic translation with the use of the rabbit reticulocyte lysate system (Seefeldt et al., 2016). Pro-rich AMPs are able to interact with several targets within bacterial cells, and therefore probability of the spontaneous resistance emergence might be rather low. The advantage of Pro-rich AMPs as compared with known conventional antibiotics targeting ribosome is an ability to simultaneously occupy several functional sites of the 50S subunit (Gagnon et al., 2016), and the modifications in rRNA does not necessarily lead to the resistance. Interestingly, mutations in the ribosome that confer resistance to erythromycin result in cross-resistance to insect Pro-rich AMPs, but not to mammalian Bac7 orthologs (Gagnon et al., 2016; Mardirossian et al., 2018). Nevertheless, the “Achilles’ heel” of most Pro-rich AMPs is the dependence on specific transport systems when getting into the bacterial cell. Moreover, an inactivation of the transport protein SbmA can reduce activity of some Pro-rich AMPs without an obvious fitness cost for the bacteria (Pränting et al., 2008). Therefore, it is surprising that many organisms produce Pro-rich AMPs to fight bacteria. The capacity for preventing resistance development appears to be a feature of the panel of AMPs as a part of whole immune system, but not of individual peptides (Chernysh et al., 2015). In particular, it is likely that membranolytic agents, e.g., α-helical amphipathic AMPs, can promote translocation of Pro-rich peptides into bacterial cell. The α-helical mammalian cathelicidins are known to have a wide spectrum of antimicrobial activity and a comparatively high toxicity as the result of moderate cell selectivity. Combined antibacterial effects between AMPs should be thoroughly investigated, as the results may explain a high efficacy of the AMP-based defense. Identification of synergistic combinations of AMPs may help to decrease effective concentrations of active molecules (Yan and Hancock, 2001), extend their spectrum of action (Lüders et al., 2003), and prevent the resistance formation (Chernysh et al., 2015). The last-mentioned could occur while using individual AMPs (Anaya-López et al., 2013). To date, only a few studies on synergy between co-localized AMPs have been performed (Singh et al., 2000; Schmitt et al., 2012).

In this study, structurally distinct goat cathelicidins – Pro-rich mini-ChBac7.5Nα and α-helical ChMAP-28 were used as the model system of defense peptides with the same localization, more specifically, in leucocytes. In contrast to the non-lytic mini-ChBac7.5Nα, cathelicidin ChMAP-28 was shown to be potent antibacterial agent with an extremely fast membrane disruption kinetics. Mini-bactenecins possess a moderate antibacterial activity which strongly depends on the ionic composition of the test medium. Thus, the presence of 0.9% NaCl results in at least several-fold decrease in the activity of mini-bactenecins against all the tested bacterial strains. The obtained data indicate a synergy between the cathelicidins against a wide range of Gram-negative bacterial species including XDR causative agents of hospital-acquired infections. Importantly, the synergistic effect was shown against Gram-negative bacteria which normally have the SbmA transport system. Earlier, it was supposed that Pro-rich AMPs cross the outer membrane of Gram-negative bacteria and then are actively transported by SbmA into the cytoplasm (Krizsan et al., 2015). Here, mini-ChBac7.5Nα was shown to effectively damage outer membrane, while the addition of 0.9% NaCl minimized the activity. Antibacterial activity of mini-bactenecins is inhibited in the presence of salt, and the electrical double layer around the cell seems to be a key barrier on the way into the cell of highly charged and relatively hydrophilic mini-ChBac7.5Nα. At the same time, ChMAP-28 can damage the outer membrane acting at nanomolar concentrations, which corresponds to fractional MICs at synergy combinations with mini-ChBac7.5Nα (see Table 3). It is important to note that the presence of mini-ChBac7.5Nα does not increase the permeability of both inner and outer membrane of *E. coli* caused by ChMAP-28. A similar effect was shown earlier when the synergy between fish histone derivatives and the membranolytic AMP pleurocidin was studied (Patrzykat et al., 2001). Taken together, the obtained data suggest that ChMAP-28 at sub-inhibitory concentrations appears to promote translocation of mini-ChBac7.5Nα into the periplasmic space. Subsequently, the Pro-rich peptide crosses the cytoplasmic membrane with the participation of specific transporters and interacts with the bacterial ribosome.

Besides, AMPs are regarded as promising drug candidates for treatment of biofilms. Complete inhibition of both planktonic and biofilm growth of clinical isolates of *E. coli* by the combination of the goat cathelicidins was indicated with the FICI value of 0.375 which validates a notable synergistic effect. According to the obtained data, synergy combinations of mammalian cathelicidins might also be perspective compounds for development of antibacterial coatings for medical biomaterials and instruments.

It is known that bacteria can become resistant to individual AMPs, that in turn could induce a cross-resistance to AMP effectors of the host innate immune system, thus compromising natural host defense against pathogens (Fleitas and Franco, 2016). The resistance problem can be solved, in particular, by application of combinations of natural AMP having a complex mechanism of antibacterial action. In this paper, capacity of the synergistic combination of the goat cathelicidins for preventing bacterial resistance is reported. Selection experiments with Pro-rich AMPs were performed earlier in low-salt media (Knappe et al., 2016; Schmidt et al., 2016). Here, we used the medium containing 0.9% NaCl. As expected, the combination was shown to keep a high activity after the 26-days selection experiment in contrast to mini-ChBac7.5Nα and the reference antibiotic polymyxin B. The 64-fold increase in the MIC value (>256 μM) was registered in the XDR *E. coli* strain subjected to selection by mini-ChBac7.5Nα just after eight initial passages. Genetic analysis of the resistant strain obtained after selection revealed the single point mutation V102E in the cytoplasmic transporter SbmA as compared with the control one. In the salt-free medium the activity of mini-ChBac7.5Nα(1–16) against this strain was decreased by 8-fold as compared with the control strain subcultured without selective pressure (see Table 5). Earlier, it was shown that the V102G strain of *E. coli* had the same lowered sensitivity to Pro-rich AMPs as the SbmA-deleted strain (Corbalan et al., 2013). Interestingly, the activity of mini-ChBac7.5Nα against the resistant strain is restored to the wild-type level in a salt-free medium that suggests an important role of the C-terminal PRPRPR fragment for translocation across cytoplasmic membrane, together with an inhibition of the bacterial translation. In *E. coli*, some Pro-rich AMPs seems to rely exclusively on the SbmA transporter system, while others, including oncocin and Bac7(1–35) were active also in the SbmA-deficient strains, likely due to the presence of another bacterial transport system coding by the *yjiL-mdtM* gene (Runti et al., 2017). Taking into account that there is no significant difference in ability of mini-ChBac7.5Nα and its shortened analog to damage bacterial cytoplasmic membrane, the presence of C-terminal hexapeptide PRPRPR could facilitate a non-lytic translocation of mini-ChBac7.5Nα or promote an interaction of the peptide with cytoplasmic transporters different from SbmA. Nevertheless, the point mutation V102E in SbmA seems to contribute but does not provide the complete resistance to mini-bactenecins (MIC of >256 μM) in the presence of salt. Moreover, the process of the resistance formation was shown to be multistage that also suggests a complexity of the acquired resistance.

Finally, the checkerboard assay was performed to evaluate the combined effects of the cathelicidins the mini-ChBac7.5Nα-resistant *E. coli* strain. The presence of ChMAP-28 at sub-inhibitory concentrations lowered the MIC of mini-ChBac7.5Nα(1–16) from >256 μM to 16 μM while the MIC of mini-ChBac7.5Nα was reduced to 1 μM, that corresponded to their individual MICs in a salt-free medium (see Table 5). This proves that at nanomolar concentrations ChMAP-28 influences outer membrane permeability, rather than damages cytoplasmic membranes of bacteria. Cell surface modifications could also prevent interactions between mini-ChBac7.5Nα and bacteria in a medium with a high ionic strength. Interestingly, the MIC values of either mini-ChBac7.5Nα or mini-ChBac7.5Nα(1–16) against the resistant *E. coli* strain are very similar to those measured in the test against *P. aeruginosa*. Also, it should be noted that we did not identify any mutations which may inactivate the SbmA protein in the clinically isolated strain *E. coli* CI 214 with a weak sensitivity to mini-ChBac7.5Nα (Supplementary Figure S4). Hereafter, it would be necessary to gain a molecular insight into the reasons of such an increase in the *E. coli* resistance to mini-bactenecins, which could be elucidated by the use of omics-based approaches.

The obtained results suggest a potential medical application of combinations of natural cathelicidins in treating of extensively drug-resistant bacterial infections. This approach will allow using a lower therapeutic dose and minimize adverse cytotoxic effects. At the same time, goat cathelicidins potentially could be used in medicine as individual agents. ChMAP-28 exhibits outstanding antibacterial properties, but being an α-helical AMP, which are known to be unstable to proteolysis, could be considered mainly as a topical antibiotic. The Pro-rich peptide mini-ChBac7.5Nα is also a perspective molecular scaffold for drug design. The resistance to Pro-rich AMPs can be overcome when administrated in a combination with a membrane active agent, in particular, with an amphipathic cationic peptide. Interestingly, the role of the antimicrobial agent in human bloodstream can be played by the α-helical cathelicidin LL-37. The murine ortholog of the peptide, designated as CRAMP, was shown to act synergistically with insect Pro-rich AMPs (Knappe et al., 2016). However, the absence of Pro-rich AMPs in human immune system as well as their ability to cross the blood–brain barrier (Stalmans et al., 2014) makes it necessary to thoroughly analyze their immunomodulatory and cytotoxic properties. Besides, a relatively low membrane activity against mammalian cells and the ability to inhibit protein biosynthesis make ribosome-targeting Pro-rich AMPs promising candidates for the development of new antitumor agents. Therefore, combined cytotoxic effects of goat cathelicidins toward mammalian cells should be investigated as well.

## Author Contributions

PP, AK, IB, AE, and SB performed the experiments. PP, AK, IB, VK, OS, AE, SB, and TO designed the experiments and analyzed data. PP, SB, and TO wrote the paper. TO contributed to the conception of the work and supervised the whole project. All authors read and approved the final manuscript.

## Conflict of Interest Statement

The authors declare that the research was conducted in the absence of any commercial or financial relationships that could be construed as a potential conflict of interest.
