# Supplementary material for: Combined Antibacterial Effects of Goat Cathelicidins With Different Mechanisms of Action
Source: Front Microbiol. 2018 Nov 30;9:2983. doi: 10.3389/fmicb.2018.02983 (PMC6284057; doi:10.3389/fmicb.2018.02983)
Supplement: Supplementary file 1 [file Data_Sheet_1.docx]

Supplementary Material

**Combined antibacterial effects of goat cathelicidins with different mechanisms of action**

Pavel V. Panteleev^1^, Ilia A. Bolosov^1^, Alexander А. Kalashnikov^1^, Vladimir N. Kokryakov^2^, Olga V. Shamova^2^, Anna A. Emelianova^1^, Sergey V. Balandin^1^, Tatiana V. Ovchinnikova^1^

*^1^*[M.M. Shemyakin](http://www.ibch.ru/about/history/personalia/740) & [Yu.A. Ovchinnikov](http://www.ibch.ru/about/history/personalia/738) Institute of Bioorganic Chemistry, the [Russian Academy of Sciences](http://www.ras.ru/), Mikhluho-Maklaya str. 16/10, Moscow, 117997, Russia

^2^Institute of Experimental Medicine, Acad. Pavlova Str. 12, Saint-Petersburg, 197376, Russia

*** Correspondence:**Tatiana V. Ovchinnikova
[ovch@ibch.ru](mailto:ovch@ibch.ru)

**Table S1.** Amino acid sequences and molecular masses of the peptides used in this study

| Peptide | Sequence | Molecular mass, Da | |
| --- | --- | --- | --- |
|  |  | Calculated [M+H]^+^ value | Measured value* |
| ChMAP-28 | GRFKRFRKKLKRLWHKVGPFVGPILHY | 3364.0 | 3364.2 |
| mini-ChBac7.5Nα | RRLRPRRPRLPRPRPRPRPRPR | 2894.8 | 2894.4 |
| mini-ChBac7.5Nα(1-16) | RRLRPRRPRLPRPRPR | 2135.4 | 2135.5 |
| Melittin | GIGAVLKVLTTGLPALISWIKRKRQQ | 2846.7 | 2846.6 |
| Tachyplesin-1 | KWCFRVCYRGICYRRCR | 2264.1 | 2263.7 |

* monoisotopic m/z were measured using MALDI-TOF MS

**Table S2.** Antibacterial activity of goat cathelicidin ChMAP-28 and last line antibiotics against XDR clinical isolates

| **Strain** | **Minimum inhibitory concentration (µM)** | | |
| --- | --- | --- | --- |
|  | **ChMAP-28** | **Polymyxin B** | **Meropenem** |
| *E. coli* (XDR CI 1057) | 0.125 | 0.125 | 0.125 |
| *E. cloacae* (XDR CI 4172) | 0.25 | 4 | 0.5 |
| *K. pneumoniae* (XDR CI 1056) | 0.125 | 0.25 | 16 |
| *A. baumanii* (XDR CI 2675) | 0.25 | 0.125 | 0.5 |
| *P. aeruginosa* (XDR CI 1049) | 0.5 | 2 | 64 |
| *P. mirabilis* (XDR CI 3423) | 0.5 | >128 | >128 |


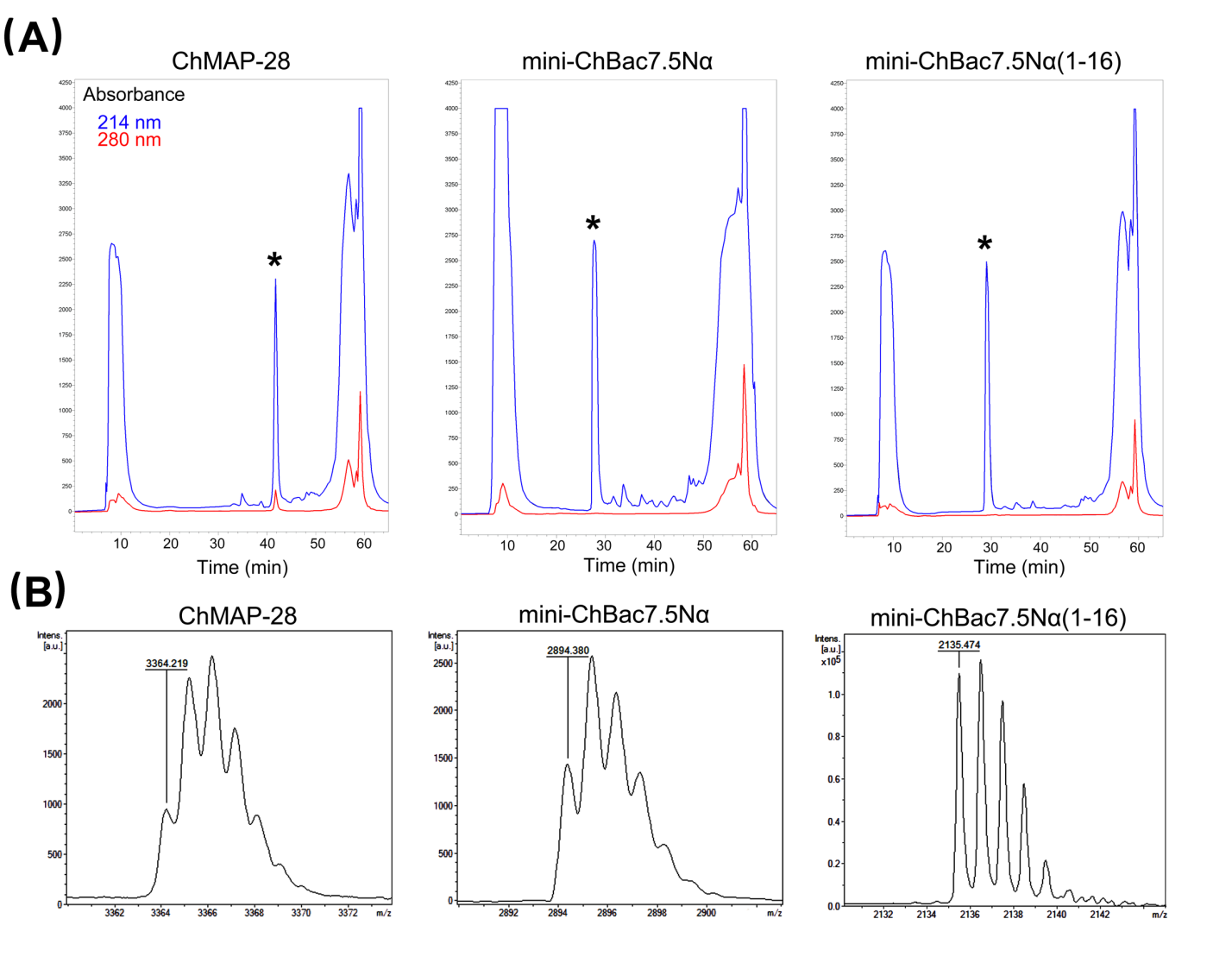


**Figure S1.** **(A)** Reversed-phase high-performance liquid chromatography (RP-HPLC) of the recombinant goat cathelicidins. RP-HPLC was performed with a linear gradient from 5 to 80% (v/v) of acetonitrile in water containing 0.1% TFA for 1 h. The mature recombinant peptide fraction is marked with an asterisk. **(B)** MALDI-MS analysis of the recombinant goat cathelicidins. The experimental [M+H]^+^ monoisotopic masses are indicated in the picture.


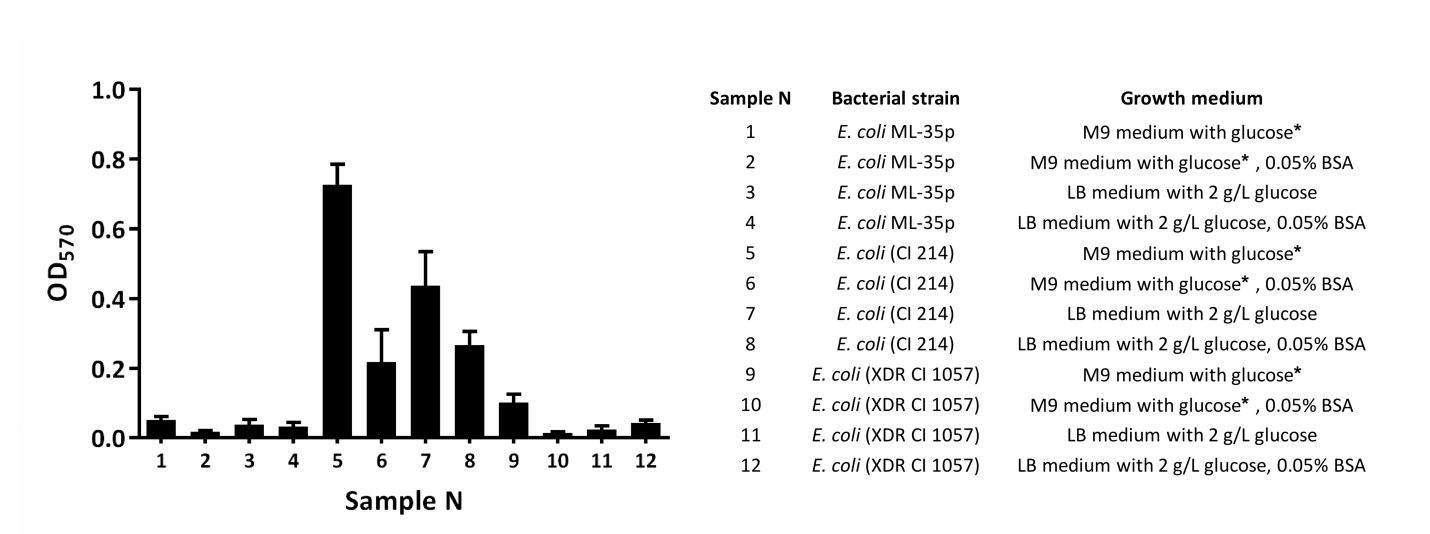


**Figure S2**. Biofilm formation capacity of tested bacteria. The *E. coli* cells were incubated in trypticase soy broth for 16 h at 37°C and then were diluted 300-fold with a corresponding medium. 100 µL of the obtained bacterial suspension were placed in 96-well flat-bottom polystyrene microplates. The plates were incubated at 32 °C with gentle agitation (120 rpm) for 24 h. Biofilm formation was assessed by the colorimetric crystal violet-based technique described in the Materials and Methods section. Data are the mean ± SD of two independent experiments performed in triplicate.

* The composition is specified in the Materials and Methods section.


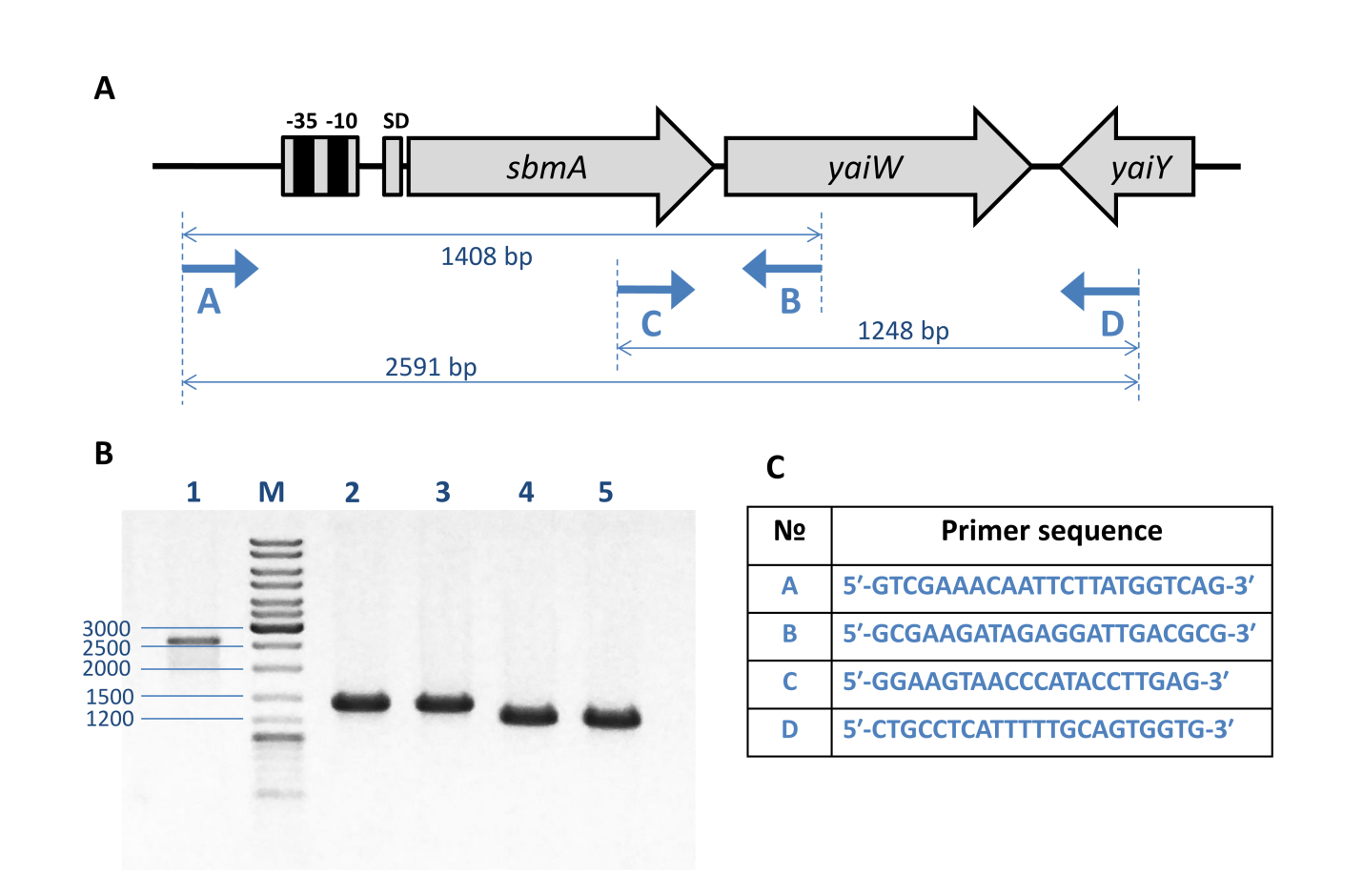


**Figure S3.** **(A)** PCR-amplification scheme of the *E. coli* *sbmA-yaiW* gene region. The *yaiW* gene, located downstream of *sbmA*, is a part of the same operon. **(B)** Agarose gel analysis of the PCR-products. Lane 1 – primers A+D: *E. coli* XDR CI 1057 (26 days with mini-ChBac7.5Nα); M – DNA molecular size marker; Lane 2 – primers A+B: *E. coli* XDR CI 1057 (wt, 26 days without antimicrobial agent); Lane 3 – primers A+B: *E. coli* XDR CI 1057 (26 days with mini-ChBac7.5Nα); Lane 4 – primers C+D: *E. coli* XDR CI 1057 (wt, 26 days without antimicrobial agent); Lane 5 – primers C+D: *E. coli* XDR CI 1057 (26 days with mini-ChBac7.5Nα). **(C)** Sequences of the primers used for PCR-amplification.


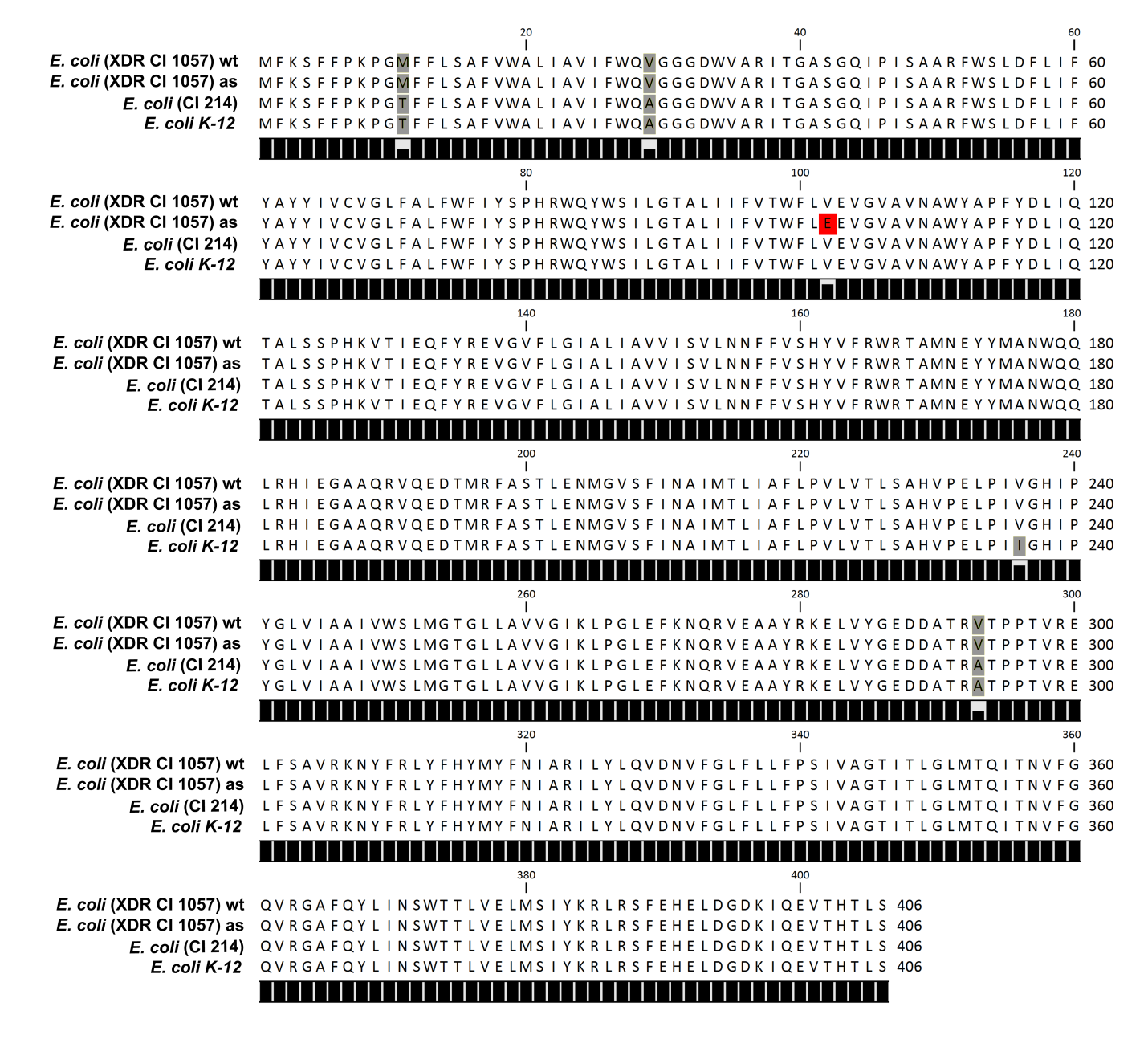


**Figure S4.** Alignment of amino acid sequences of SbmA from *E. coli* XDR CI 1057 “wt” (wild type, 26 days without antimicrobial agent), from mini-ChBac7.5Nα-resistant *E. coli* XDR CI 1057 **“**as” (after selection, 26 days with mini-ChBac7.5Nα), *E. coli* (CI 214) with a weak sensitivity to mini-ChBac7.5Nα, and the reference strain *E. coli* K-12 (Genbank access number AAC73480.1). Amino acid substitution V102E in SbmA protein is highlighted with red.
